# A Novel Quality-Control Procedure to Improve the Accuracy of Rare Variant Calling in SNP Arrays

**DOI:** 10.3389/fgene.2021.736390

**Published:** 2021-10-26

**Authors:** Ting-Hsuan Sun, Yu-Hsuan Joni Shao, Chien-Lin Mao, Miao-Neng Hung, Yi-Yun Lo, Tai-Ming Ko, Tzu-Hung Hsiao

**Affiliations:** ^1^ Department of Biological Science and Technology, National Yang Ming Chiao Tung University, Hsinchu, Taiwan; ^2^ Department of Medical Research, Taichung Veterans General Hospital, Taichung, Taiwan; ^3^ Graduate Institute of Biomedical Informatics, College of Medical Science and Technology, Taipei Medical University, Taipei, Taiwan; ^4^ Clinical Big Data Research Center, Taipei Medical University Hospital, Taipei, Taiwan; ^5^ Institute of Biomedical Sciences, Academia Sinica, Taipei, Taiwan; ^6^ Department of Public Health, Fu Jen Catholic University, New Taipei City, Taiwan; ^7^ Institute of Genomics and Bioinformatics, National Chung Hsing University, Taichung, Taiwan; ^8^ Research Center for Biomedical Science and Engineering, National Tsing Hua University, Hsinchu, Taiwan

**Keywords:** SNP array, rare variant, quality-control, genotyping, disease screening

## Abstract

**Background:** Single-nucleotide polymorphism (SNP) arrays are an ideal technology for genotyping genetic variants in mass screening. However, using SNP arrays to detect rare variants [with a minor allele frequency (MAF) of <1%] is still a challenge because of noise signals and batch effects. An approach that improves the genotyping quality is needed for clinical applications.

**Methods:** We developed a quality-control procedure for rare variants which integrates different algorithms, filters, and experiments to increase the accuracy of variant calling. Using data from the TWB 2.0 custom Axiom array, we adopted an advanced normalization adjustment to prevent false calls caused by splitting the cluster and a rare het adjustment which decreases false calls in rare variants. The concordance of allelic frequencies from array data was compared to those from sequencing datasets of Taiwanese. Finally, genotyping results were used to detect familial hypercholesterolemia (FH), thrombophilia (TH), and maturity-onset diabetes of the young (MODY) to assess the performance in disease screening. All heterozygous calls were verified by Sanger sequencing or qPCR. The positive predictive value (PPV) of each step was estimated to evaluate the performance of our procedure.

**Results:** We analyzed SNP array data from 43,433 individuals, which interrogated 267,247 rare variants. The advanced normalization and rare het adjustment methods adjusted genotyping calling of 168,134 variants (96.49%). We further removed 3916 probesets which were discordant in MAFs between the SNP array and sequencing data. The PPV for detecting pathogenic variants with 0.01%<MAF≤1% exceeded 99.37%. PPVs for those with an MAF of ≤0.01% improved from 95% to 100% for FH, 42.11% to 85.19% for TH, and 18.24% to 72.22% for MODY after adopting our rare variant quality-control procedure and experimental verification.

**Conclusion:** Adopting our quality-control procedure, SNP arrays can adequately detect variants with MAF values ranging 0.01%∼0.1%. For variants with MAF values of ≤0.01%, experimental validation is needed unless sequencing data from a homogeneous population of >10,000 are available. The results demonstrated our procedure could perform correct genotype calling of rare variants. It provides a solution of pathogenic variant detection through SNP array. The approach brings tremendous promise for implementing precision medicine in medical practice.

## Introduction

Globally, over 7,000 rare diseases affect 5–10% of the population (Richmond et al., 2021). Most of these diseases are caused by rare pathogenic variants, which have a minor allele frequency (MAF) in a population of <1% with high penetrance (Gautheron and Jéru, 2020). For example, inherited retinal degenerations are caused by mutations of 271 genes, including the EYS and ABCB4 genes (Chen et al., 2021; Retnet, (1996). Recent studies also discovered several pathogenic variants which are associated with common traits or complex diseases, such as hyperlipidemia, myocardial infarction, and diabetes (Lee et al., 2014; Riddle et al., 2020; Vrablik et al., 2020; Momozawa and Mizukami, 2021). Although detecting rare variants is important, it is still challenged because of the low MAF values. Large-scale genomic data are needed to identify pathogenic variants with a large effect and high penetrance.

Recently, several biobanks have been set up and have collected large-scale genetic data, including single-nucleotide polymorphism (SNP) arrays and next-generation sequencing (NGS) data (Chen et al., 2011; Bycroft et al., 2018; Kanai et al., 2018). Through such data, many rare pathogenic variants have been identified (Cirulli et al., 2020), (Blauwendraat et al., 2021) and have been widely used as disease-associated genetic markers, including monogenic (Firdous et al., 2018) and complex diseases (Marvel et al., 2017; Jurgens, 2020; Patel et al., 2020; Chen et al., 2021). High-density SNP arrays provide a rapid and efficient method to simultaneously genotype hundreds of thousands of specific variants (Kim and Misra, 2007; Visscher et al., 2017; Kim et al., 2018). The Taiwan Biobank has utilized SNP arrays to discover specific variants associated with hereditary diseases, drug metabolism, and drug responses of the Han Chinese population in Taiwan (Lin et al., 2019). Also, several companies, such as 23andme, provide direct-to-consumer genetic testing services which assess genetic risks for diseases or health conditions using SNP arrays (Tandy-Connor et al., 2018; Horton et al., 2019; Schleit et al., 2019). However, recent articles have pointed out the low accuracy and high false positive rates of SNP arrays for detecting rare variants (Wright et al., 2019; BMJ, 2021).

Variant calling of SNP arrays relies on clustering of probeset signals (Lamy et al., 2006). Clustering of rare variants becomes very difficult when only a limited number of alternative alleles exist (Weedon, 2019). Differences in signal distributions due to batch effects also cause misclustering. As shown as Supplementary Figure S1 in “Supplemental materials”, samples in a batch with high average signals of major alleles can be misclassified as alternative alleles. Noise signals induced in the experiment, such as by air bubbles or scratches, also cause incorrect calling (Supplementary Figure S1B). In addition, cross-hybridization reactions with non-target sequences can also induce false calling for probesets with low specificity for targeting sequences. The performance of probesets for rare variants is difficult to evaluate because of the low frequency of alternative alleles. Although several algorithms or procedures have been developed to improve the accuracy of genotyping of common variants (Hua et al., 2007; Xiao et al., 2007; Hunter-Zinck et al., 2020), methods that focus on rare variant calling for SNP arrays are still lacking. New strategies are needed to improve the accuracy of rare variants for further applications.

The objective of this study is to develop a rare variant quality-control (QC) procedure to improve the calling accuracy. We proposed a procedure combining advance normalization, rare het adjustment, and MAF comparisons to improve true positive rate. This approach was evaluated by Sanger validation or real-time Polymerase Chain Reaction (qPCR) and an external data set. As we demonstrate, our method provides a solution which makes SNP arrays feasible as screening tools for rare variants.

## Materials and Methods

### Dataset of Single-Nucleotide Polymorphism Arrays

We used SNP arrays from the project of Taiwan Precision Medicine Initiative (TPMI) to conduct our data analysis. In total, 43,531 individuals were recruited from Taichung Veterans General Hospital (TCVGH; Taichung, Taiwan). Participants consented for blood to be drawn and SNP arrays to be performed, as well as for their clinical information to be linked. DNA of participants was extracted for genotyping on a custom Axiom array, TWB2.0, which was designed by the TWB based on 970 whole-genome sequence (WGS) data in the Taiwanese population (Lin et al., 2019).

In total, 714,461 probesets were designed on the TWB 2.0 Array plate (Santa Clara, CA, United States). It contained about 415,000 probesets for gene-wide association studies (GWASs) and imputation and also about 114,000 probesets for risk or pathogenic analysis selected from several sources, including ACMG, ClinVar, GWAS Catalog, HGMD, and the literature. We selected 267,247 probe-sets associated with rare variants (with an MAF of <1% in NGS data) to evaluate the calling accuracy of rare variants.

### Genotype Calling, Advanced Normalization, and Rare Het Adjustment

Genotype calling was based on Affymetrix^®^ Power Tools (APT, command-line software, Santa Clara, CA, United States). Data of 24 plates were grouped as a batch according to the processing date. After genotyping, we applied advanced normalization to adjust misclustering based on the batch effect. Advanced normalization was conducted with the advnorm package provided by Thermo Fisher Scientific (Santa Clara, CA, United States). We also applied a rare het adjustment to exclude probesets with different signals in replicated probes. The rare het adjustment was conducted with the axiomBestPractices-1.2.4 program and the command “-do-rare-het-adjustment’’ (see supplemental methods).

### Comparing Allelic Frequencies Between the Single-Nucleotide Polymorphism Array and NGS Data

We collected 3,370 WGSs to estimate the MAFs for all variants. Data of 1,200 WGSs were accessed from TWB, and 2,170 of them were collected from our in-house program. Briefly, about 30× whole-genome sequencing was performed. Reads were aligned to the GRCh38 human genome using the GATK pipeline. Values of the MAF were calculated using VCFtools.

To compare allelic frequencies, MAFs between the SNP array and sequencing data were log_2_ transformed. Log_2_ ratios of MAFs between the SNP array and sequencing data were calculated and utilized as parameters to estimate the MAP concordance. With the predict method Setting the upper and lower thresholds as 1.72 and −2, respectively, the probe-sets with mis-concordance of MAFs were identified and excluded.

### Variant Validation Based on a Quantitative Polymerase Chain Reaction (qPCR) or Sanger Sequencing

We respectively selected 1,090, 55, and 132 probesets of familial hypercholesterolemia (FH), thrombophilia (TH), and maturity-onset diabetes of the young (MODY) for disease-oriented analyses. The MAF distributions of the probesets are shown in Supplementary Table S2 in “Supplemental materials”. All samples with heterozygous calls were validated by a qPCR or Sanger sequencing. We used the Applied Biosystems™ Primer Designer™ Tool (Applied Biosystems, Santa Clara, CA, United States) to pick specific primer pairs for Sanger sequencing, we designed primers with the Primer3 algorithm (Kumar and Chordia, 2015) and checked for sequence similarities throughout the human genome using the Primer-BLAST tool (Ye et al., 2012).

### Independent Dataset for External Validation

Newly collected SNP array data of 5,358 samples was used to measure the reproducibility of our QC procedure. We used the same workflow to genotype calling. Samples with heterozygous calls were verified through qPCR or Sanger sequencing to evaluate calling accuracy.

## Results

### An Analytical Algorithm for Rare Variant Detection

**FIGURE 1:**
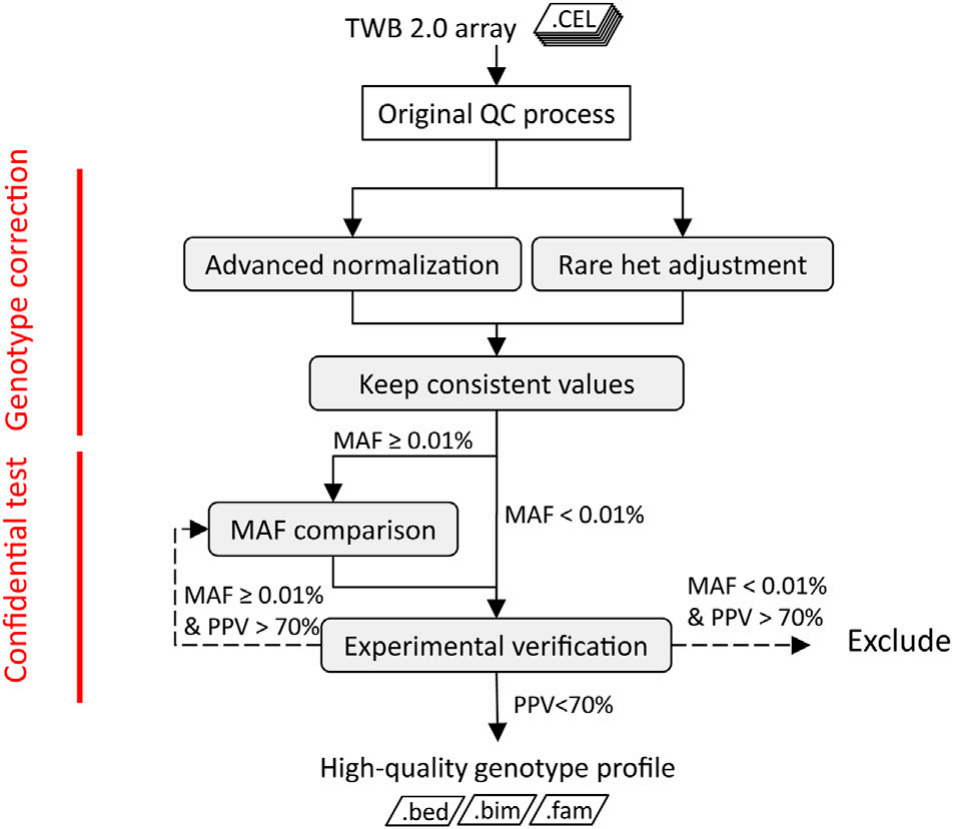
Quality-control procedure of TWB 2.0 arrays of rare variants. The shaded boxes are steps of the rare variant quality-control procedure. The dotted line represents the management of variants with low PPV.

We implemented a QC procedure that contains four key components to precisely detect rare variants in SNP arrays. As shown in Figure 1A, two algorithms, advanced normalization and rare het adjustment, were applied to post-QC data. The rare het adjustment checked signals of heterozygous calls from each replicate probe group and compared the signal distribution in a batch. Heterozygous calls were adjusted to “no call” if the signal distribution from the replicate probe group was uncertain. Advanced normalization detected clustering errors from specific plates in batches and reassigned those calls to the correct cluster. We consolidated results from the two algorithms and filtered conflicting results. In the third step, we compared the concordance of the MAF of each variant in the array with the corresponding variant in Taiwanese WGS data. Probesets with high deviations of MAF noted as low-concordance probes were excluded from the following analysis. Last, we assessed the performance of the genotyping results in disease screening and verified the results by a qPCR or Sanger. We designed this integrating approach to improve the quality of SNP array data in genotyping rare variants.

### Genotype Correction Procedure Adjusts Incorrect Calls on the Array

**FIGURE 2:**
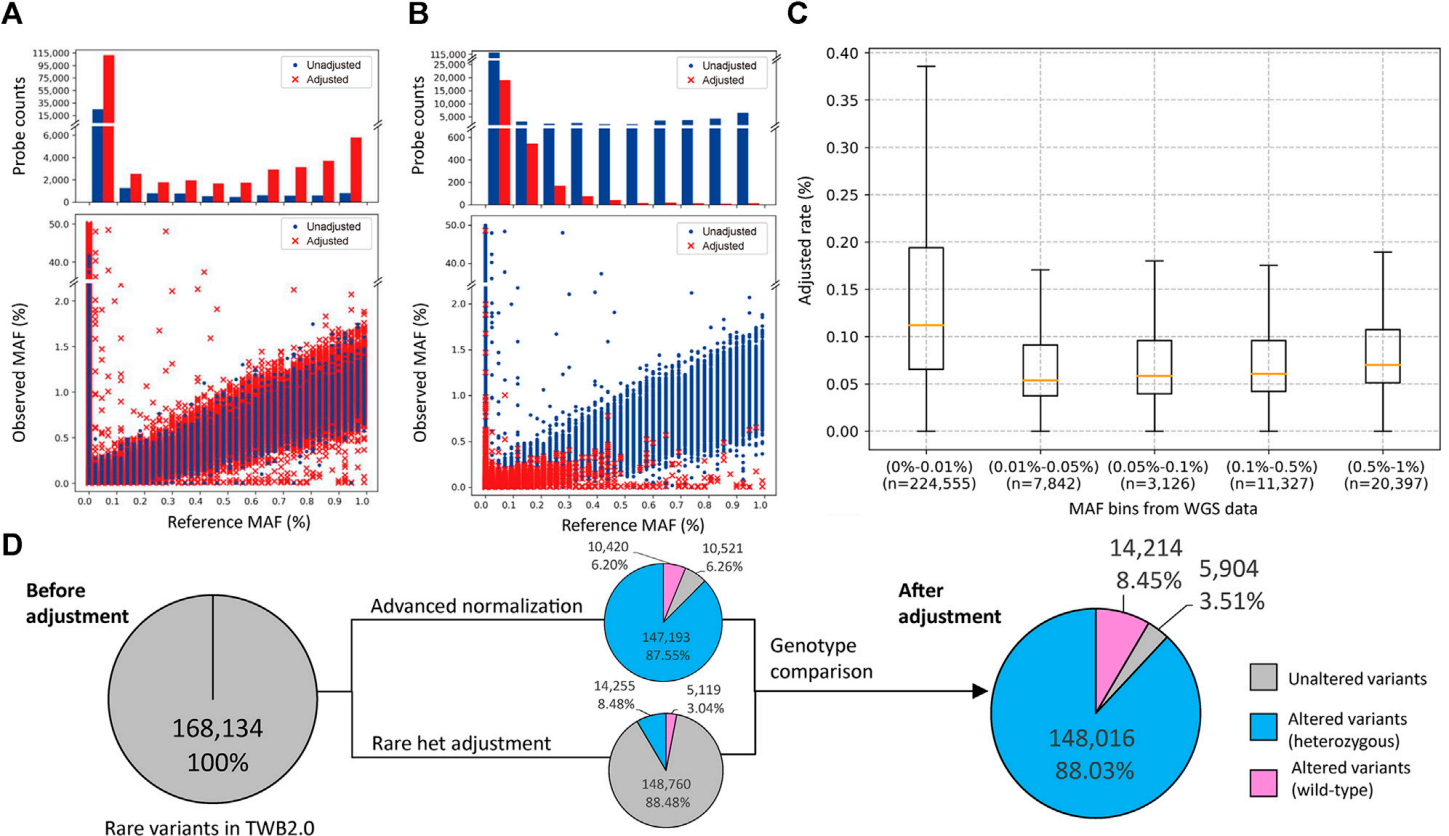
Minor allele frequency (MAF) distribution and probeset count after performance of the rare variant quality-control procedure. **(A,B)** The former is the MAF distribution after using the advanced normalization method, and the latter is the MAF distribution after using the rare het adjustment method. The upper plot shows the number of probesets adjusted (red bar) or unadjusted (blue bar) by this algorithm. The bottom plot shows the distribution of MAFs between the single-nucleotide polymorphism (SNP) array and sequencing data. Adjusted data points are marked with a red “×”, and unadjusted data points are marked with a blue dot. **(C)** Box plot of the adjusted rate per MAF bin based on sequencing data. **(D)** Number of rare variants with at least one heterozygous call in the original data, and after advanced normalization and rare het adjustment. Variants not adjusted by our algorithms are represented as a gray slice. Variants adjusted by our algorithms are represented as a blue slice. Variants adjusted by our algorithms are represented as a pink slice.

We analyzed SNP array data from 43,433 individuals, which interrogated 267,247 rare variants. We assessed the crude number and rate of adjustments made by the two algorithms in correlation with the MAF. We adjusted 136,773 calls with the advanced normalization method (Figure 2A) and 19,347 calls with the rare het adjustment method (Figure 2B). Figure 2C shows distributions of variants that were adjusted by MAF. The adjustment rate was the highest in variants with MAFs of ≤0.01%.

In these genotype callings, 168,134 variants had at least one heterozygous call, and 99,110 variants were completely wild-type. The advanced normalization algorithm adjusted 10,420 variants (6.20%) to be the wild-type and modified the number of heterozygous calls in 147,193 variants (87.55%). The rare het adjustment algorithm adjusted 5,119 variants (3.04%) to be the wild-type and modified the number of heterozygous calls in 14,255 variants (8.48%). Taken together, our algorithm adjusted the genotyping calling of 162,230 variants (96.49%) in which 14,214 variants (8.45%) were adjusted to the wild-type and 148,016 variants (88.03%) were modified. Only 5,904 variants (3.51%) remained the same after these adjustments (Figure 2D).

### Discordance by Minor Allele Frequencys

**FIGURE 3:**
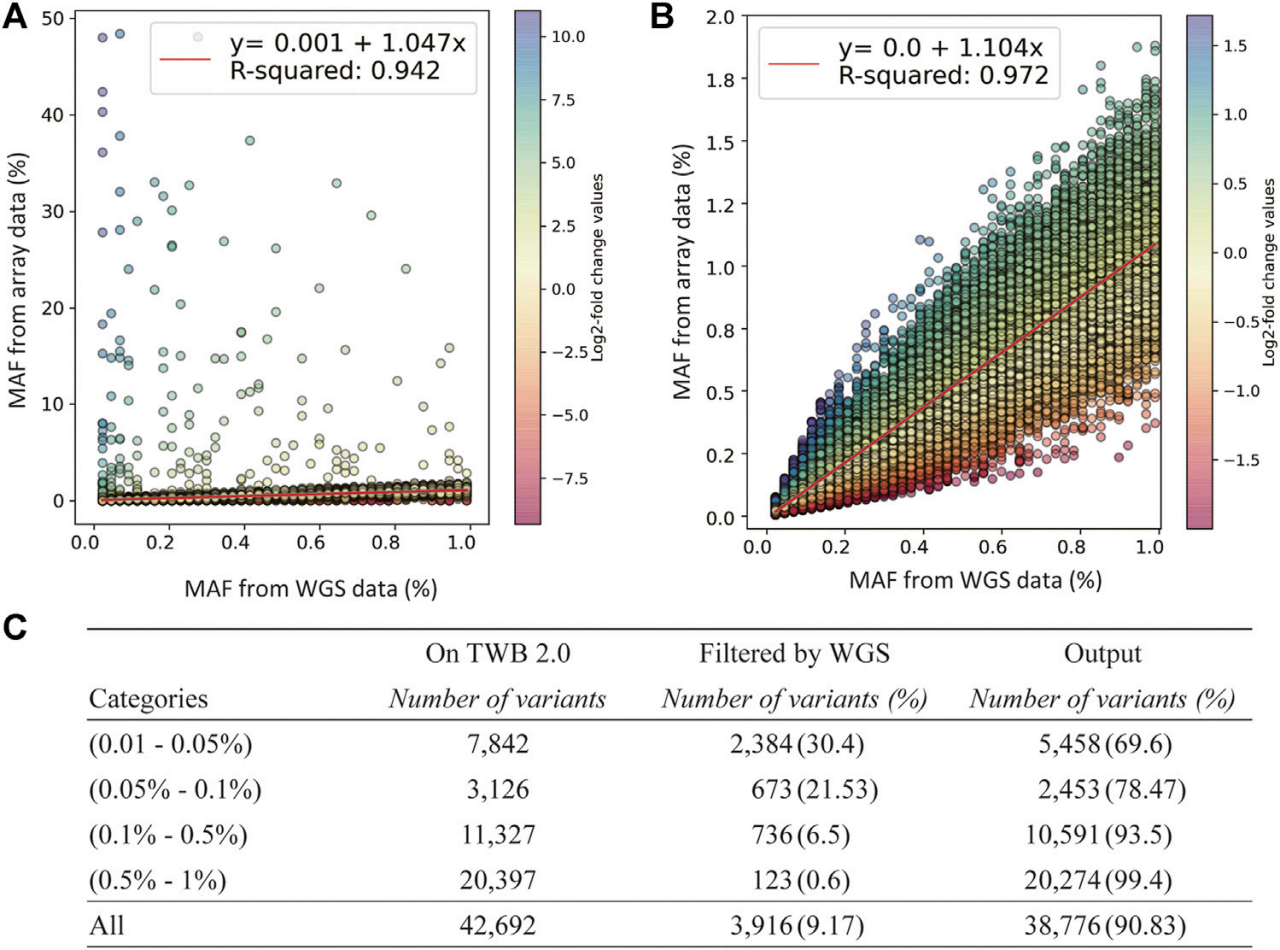
Quality-control assessments on the TWB 2.0 data after performance of rare variant quality-control procedures. **(A)** Comparison of minor allele frequencies (MAFs) between sequencing data and the TWB 2.0 before excluding low-concordance probes. **(B)** Comparison of MAFs between sequencing data and the TWB 2.0 after excluding low-concordance probes. **(C)** The distribution of excluded probes according to the MAF bins.

We used log2 ratio of the computed MAFs as the parameter to compare between the SNP array and NGS (Figure 3A). By setting the upper threshold value to 1.72 and lower to −2, we were able to obtain the highest coefficient of determination in the MAF scatter plot. We removed 3,916 low-concordance probe-sets which were discordant in MAFs between the SNP array and NGS. The remaining probesets showed good concordance. The coefficient of the linear regression was 1.104, and the coefficient of determination was 0.972 (Figure 3B).

The number of discordant probesets in each MAF group is presented in Figure 3C. Compared to 0.6% of the probesets in the group of 0.5% < MAF≤1% which showed low concordance, 30.4% of the probesets in the group of 0.05% < MAF≤0.01% showed low concordance. This step excluded lots of non-working probesets. However, variants in the group of MAF<0.01% could not be applied due to limited MAF resolution of sequencing data.

### Disease Screening and Experimental Validation of the True Positive Rate

To test the performance of genotype calling in disease screening after applying our analytical procedure, we investigated pathogenic variants of three hereditary diseases: FH, TH, and MODY. Totals of 1,090, 132, and 55 pathogenic variants on the SNP array were respectively associated with FH, MODY, and TH. There were 499 mutation carriers detected in FH, 76 in TH, and 148 in MODY in the original data. Numbers of carriers are presented in Table 1 by 0.01% < MAF≤1% and MAF≤0.01%. We verified all samples with heterozygous genotypes through Sanger sequencing or a qPCR. Using our rare-variant QC procedure and experimental verification, the PPV improved from 98.96 to 99.37% in variants with 0.01% < MAF≤1% and from 95 to 100% in variants with MAF≤0.01% in FH. The PPV remained 100% in variants with 0.01% < MAF≤1%, and it improved from 42.11 to 85.19% in variants with MAF≤0.01% in TH. In detecting pathogenic variants in MODY, all variants were in the group of MAF≤0.01%, and the PPV improved from 18.24 to 72.22% after our QC approach. We also examined 1.35% of negative calls and reached 100% of negative predictive value (NPV). The filtration trace in each step is shown in Supplementary Table S3.

**TABLE 1:** Performance of the TWB 2.0 in detecting rare pathogenic variants for familial hypercholesterolemia, thrombophilia, and maturity onset diabetes of the young in data with the original quality-control process, with rare variants quality-control procedure, and experimental verification.

	Familial hypercholesterolemia						Thrombophilia						Maturity-onset diabetes of the young					
—	0.01% < MAF≤1%			MAF≤0.01%			0.01% < MAF≤1%			MAF≤0.01%			0.01% < MAF≤1%			MAF≤0.01%		
Dataset	TPs	FPs	PPV (%)	TPs	FPs	PPV (%)	TPs	FPs	PPV (%)	TPs	FPs	PPV (%)	TPs	FPs	PPV (%)	TPs	FPs	PPV (%)
Original	474	5	98.96	19	1	95.00	19	0	100	24	33	42.11	—	—	—	27	121	18.24
With rare variants QC	470	3	99.37	17	0	100	19	0	100	23	4	85.19	—	—	—	26	10	72.22
External dataset (5,358 samples)	67	1	98.53	2	0	100	2	0	100	0	1	0	—	—	—	2	0	100

MAF, minor allele frequency; TPs, true positives; FPs, false positives; PPV positive predictive value.

The qPCR and Sanger sequencing results are the gold standard for TP and FP.

In addition, we used an independent dataset of 5,358 samples to evaluate our procedures as an external validation. We reached 98.57, 100 and 66.67% of positive predictive value in familial hypercholesterolemia (FH), thrombophilia (TH), and maturity-onset diabetes of the young (MODY) respectively. (Table 1)

## Discussion

SNP array was widely used for variant calling. This proposed quality-control procedure can improve the accuracy of rare variant calling to extend the application of SNP array. Although NGS has high accuracy of rare variant detection and considered as the gold standard, it requires high computational power and skilled bioinformaticians for variant calling. The computational process is massive when we have a large number of samples. SNP array is an alternative method to detect known-pathogenic variants. It is more convenient and efficient for variant calling due to the characteristic of probe hybridization comparing to WGS. This procedure makes SNP array suitable for pathogenic variant screening. Also, it enables us to utilize available biobank data for studying pathogenic variant in a population level.

We developed a robust QC pipeline which can effectively adjust false positive calling in rare variants and increase the positive detection rate. Two major algorithms, rare het adjustment and advance normalization, were used to correct false signals, while MAF comparisons tagged low-concordance probesets. Our data showed dramatic improvements in true positive rates. The PPVs for MODY and TH improved from 18 to 93% and from 57 to 100%, respectively. The demonstrated performance indicated that SNP array data combined with our QC algorithm could be directly applied to large-scale disease screening for the Taiwanese population.

The data of the original genotype calling algorithm showed a poor performance for rare variants in some cases. Positive rates of TH and MODY were as low as 56.58 and 17.53%, respectively. One of the potential reasons is that the original genotyping pipeline was designed for common variants (Bush and Moore, 2012). Most rare variants lack alternative alleles, causing an extremely skewed dataset for initial genotype gating and cluster splitting. This leads to incorrect genotype calling (King and Nicolae, 2014). Another reason for false calling could be induced by abnormal fluorescence signals due to bubbles or scratches. Although most probesets of the SNP array were designed for repetitive probes and randomly distributed at different locations to eliminate the effect, extremely high signals from bubbles or scratches will increase the average signal and raise false calls (BioRxiv, 2020). We introduced the rare het adjustment to eliminate this effect. It changed the unexpected result to “no call”. Wrong clustering caused by batch effects is another source of incorrect calling. This kind of probeset often has a high intensity in the genotype cluster plot, leading to a missed split into the wrong cluster. To target this issue, advanced normalization was applied to identify batch effects and reassign genotype clusters. As the result we demonstrated, the two algorithms increased the PPVs of TH and MODY. By combining the two procedures, the performance of base calling of rare variants could be dramatically increased to 91.30 and 72.22%, respectively.

Incorrect calls from low-concordance probes are an important issue in rare variant detection. They can be caused by an improper probe design and non-specific hybridization. We utilized the procedure of MAF comparison to check the concordance of MAFs between array data and sequencing. Any probes with out-of-range MAF values were identified as low-concordance probes and excluded. This procedure marked 9.17% of probesets with low performance in the array. However, the procedure only works on variants with a frequency of >0.01% due the resolution of sequencing data. For probesets that interrogated variants with a frequency of <0.01%, experimental validation was used to test the concordance of the probesets.

Our data reveal the challenge of detecting rare mutations, especially of variants with a frequency of <0.01%. The positive rate decreased when the MAF decreased. For example, the positive detection rate of FH was up to 98.8% in the original data. MAFs of FH variants were mainly in the range of 0.5–0.01%. However, the positive rate of MODY was down to 17.53%, because the frequency of all variants was <0.01%. By considering the issue discussed above, our procedure demonstrated significant improvement in the positive prediction rate without losing many true positive calls, but one positive call was lost for TH and MODY.

We used the Log_2_ ratios of MAFs between the SNP array and sequencing data as a parameter to estimate the MAF concordance. Ideally, the coefficient of regression line should be 1 if the MAFs of SNP array and sequencing are came from the same samples. However, the MAFs of SNP data and sequence data are derived from different cohort in our study. We observed the difference between array MAFs and WGS MAFs. Taking two variants we did experimental validation as example, the MAF of rs749038326 is 0.0842% in SNP array, but it is 0.0461% in the sequencing data. The MAF of another SNP, rs730882109, is 0.192% in the array data, but it is 0.0691% in the sequencing data. Few reasons can help to explain this phenomenon. First, the SNP data and WES data were derived from different cohorts. Different age distribution and disease condition of two cohorts may cause the discrepancy. In addition, the total number in the SNP data and the WES data are different. MAFs estimated from WGS did not provide enough resolution for rare variants.

As increasing numbers of novel variants are discovered from sequencing and WGS approaches, custom-designed genotyping arrays are an alternative strategy for investigating low-frequency and rare variants for large cohorts with the advantage of low costs (Hurd and Nelson, 2009; Berry et al., 2019). It The procedure we developed provides an excellent solution to overcome genotyping call issues in rare variants. In addition, as results we demonstrated, SNP arrays can be used for genetic disease screening. This has great potential for clinical utilization based on the advantage of low costs and low demands for computational power. The capacity of SNP arrays is up to millions of SNPs. All known pathogenic variants of genetic diseases in populations could be screened simultaneously. Our procedure provides a solution of correct genotype calling. Combined together, the approach brings tremendous promise for implementing precision medicine in medical practice.

## Data Availability

The SNP array data in this study is from TPMI projects (https://tpmi.ibms.sinica.edu.tw/www/en/). A portion of the WGS data are from Taiwan BioBank (https://www.twbiobank.org.tw/new_web_en/about-export.php). The data analyzed in this study is subject to the following licenses/restrictions: One can apply to access Taiwan Biobank Data. Requests to access these datasets should be directed to biobank@gate.sinica.edu.tw.
