# Supplementary material for: A Novel Quality-Control Procedure to Improve the Accuracy of Rare Variant Calling in SNP Arrays
Source: Front Genet. 2021 Oct 26;12:736390. doi: 10.3389/fgene.2021.736390 (PMC8577504; doi:10.3389/fgene.2021.736390)
Supplement: Supplementary file 1 [file DataSheet1.docx]

**A novel quality-control procedure to improve the accuracy of rare variant calling in SNP arrays**

Ting-Hsuan Sun, Yu-Hsuan Shao^*^, Chien-Lin Mao, Miao-Neng Hung, Yi-Yun Lo, Tai-Ming Ko, and Tzu-Hung Hsiao^*^

**Supplemental Information**

**Supplemental Figure 1. Genotyping results before and after algorithm adjustments.**

1. Advanced normalization. B. Rare het adjustment.


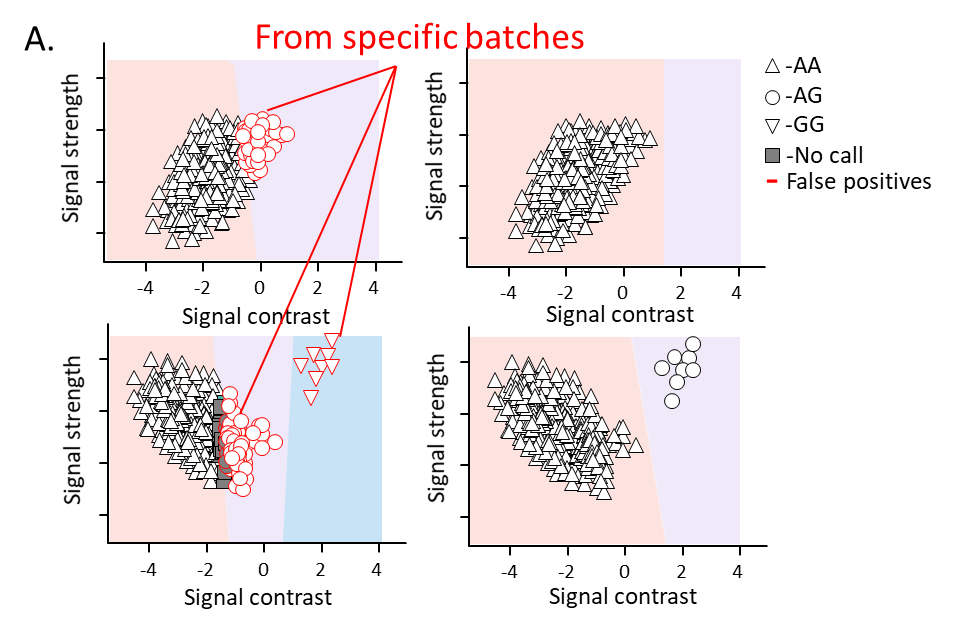


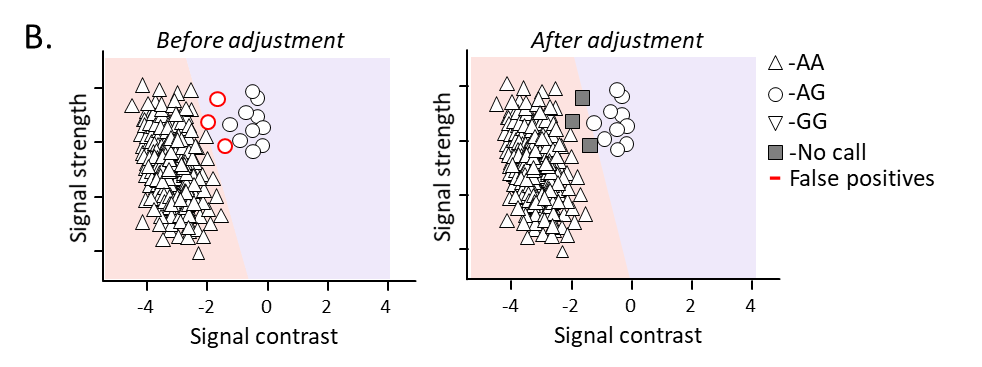


**Supplemental table 1. The proportion of probe sets and variants classified into the various minor allele frequency (MAF) bins**

B.

Signal strength

Signal contrast

-4

-2

0

2

4

*Before adjustment*

Signal strength

Signal contrast

-4

-2

0

2

4

*After adjustment*

False positives

-No call

-GG

-AA

-AG

|  | MAF bin | No. of probes | No. of SNPs |
| --- | --- | --- | --- |
| Rare variants | (0%~0.01%) | 224,555 | 221,582 |
|  | (0.01%~0.05%) | 7842 | 7841 |
|  | (0.05%~0.1%) | 3126 | 3126 |
|  | (0.1%~0.5%) | 11,327 | 11,327 |
|  | (0.5%~1%) | 20,397 | 20,397 |
| Low-frequency variants | (1%~5%) | 188,658 | 188,658 |
| Common variants | (5%~100%) | 216,622 | 216,619 |
|  | All | 672,527 | 669,550 |

**Supplemental table 2. The number of probe sets that detected disease-related variants in the minor allele frequency (MAF) bins.**

FH, familial hypercholesterolemia; TH, thrombophilia; MODY, maturity-onset diabetes of the young.

| Category | FH | TH | MODY |
| --- | --- | --- | --- |
| (0%~0.01%) | 1079 | 54 | 130 |
| (0.01%~0.05%) | 5 |  | 2 |
| (0.05%~0.1%) | 3 | 1 |  |
| (0.1%~0.5%) | 3 |  |  |
| All | 1090 | 55 | 132 |

**Supplemental table 3. The filtration trace of each probe**

| **DIS** | **rsID** | **CHR** | **POS** | **Alt** | **Ref** | **Gene** | **Rare het  adjustment** | **Advance  normalization** | **Genotype  comparison** | **Filter with  WGS MAF** | **Experimental  verification** | **MAF_WGS** | **MAF bin** |
| --- | --- | --- | --- | --- | --- | --- | --- | --- | --- | --- | --- | --- | --- |
| FH | rs144467873 | chr2 | 21006289 | A | G | APOB | KEEP | KEEP | KEEP | KEEP | KEEP | 1.84E-03 | 0.001%~0.005% |
| FH | rs769446356 | chr19 | 11100349 | T | A | LDLR | KEEP | KEEP | KEEP | KEEP | KEEP | 1.15E-03 | 0.001%~0.005% |
| FH | rs730882109 | chr19 | 11116900 | T | C | LDLR | KEEP | KEEP | KEEP | KEEP | KEEP | 6.91E-04 | 0.0005%~0.001% |
| FH | rs749038326 | chr19 | 11102741 | A | G | LDLR | KEEP | KEEP | KEEP | KEEP | KEEP | 4.61E-04 | 0.0001%~0.0005% |
| FH | rs5742904 | chr2 | 21006288 | T | C | APOB | KEEP | KEEP | KEEP | KEEP | KEEP | 0.00E+00 | 0%~0.0001% |
| FH | rs875989921 | chr19 | 11113624 | A | G | LDLR | KEEP | KEEP | KEEP | KEEP | KEEP | 0.00E+00 | 0%~0.0001% |
| FH | rs28942084 | chr19 | 11120436 | T | C | LDLR | KEEP | KEEP | KEEP | KEEP | KEEP | 0.00E+00 | 0%~0.0001% |
| MODY | rs193922339 | chr7 | 44146535 | T | A | GCK | KEEP | KEEP | KEEP | KEEP | KEEP | 0.00E+00 | 0%~0.0001% |
| MODY | rs267607196 | chr11 | 17387248 | T | C | KCNJ11 | KEEP | KEEP | KEEP | KEEP | KEEP | 0.00E+00 | 0%~0.0001% |
| MODY | rs774637975 | chr12 | 120978797 | T | C | HNF1A-AS1;HNF1A | KEEP | KEEP | KEEP | KEEP | KEEP | 0.00E+00 | 0%~0.0001% |
| MODY | rs587780357 | chr12 | 120993601 | A | G | HNF1A | KEEP | KEEP | KEEP | KEEP | KEEP | 0.00E+00 | 0%~0.0001% |
| MODY | rs193921338 | chr7 | 44145590 | A | G | GCK | KEEP | KEEP | KEEP | KEEP | KEEP | 0.00E+00 | 0%~0.0001% |
| MODY | rs1375656631 | chr7 | 44147738 | T | C | GCK | KEEP | KEEP | KEEP | KEEP | KEEP | 0.00E+00 | 0%~0.0001% |
| MODY | rs1554924540 | chr11 | 17427866 | A | C | ABCC8 | KEEP | KEEP | KEEP | KEEP | KEEP | 0.00E+00 | 0%~0.0001% |
| MODY | rs1085307913 | chr20 | 44413709 | A | G | HNF4A | KEEP | KEEP | KEEP | KEEP | KEEP | 0.00E+00 | 0%~0.0001% |
| Thrombophilia | rs121909569 | chr1 | 173911981 | G | A | SERPINC1 | KEEP | KEEP | KEEP | KEEP | KEEP | 9.22E-04 | 0.0005%~0.001% |
| Thrombophilia | rs6025 | chr1 | 169549811 | T | C | F5 | KEEP | KEEP | KEEP | KEEP | KEEP | 0.00E+00 | 0%~0.0001% |
| Thrombophilia | rs121909570 | chr1 | 173911923 | G | T | SERPINC1 | KEEP | KEEP | KEEP | KEEP | KEEP | 0.00E+00 | 0%~0.0001% |
| Thrombophilia | rs121918150 | chr2 | 127428560 | A | G | PROC | KEEP | KEEP | KEEP | KEEP | KEEP | 0.00E+00 | 0%~0.0001% |
| Thrombophilia | rs387906674 | chr3 | 93893025 | A | G | PROS1 | KEEP | KEEP | KEEP | KEEP | KEEP | 0.00E+00 | 0%~0.0001% |
| FH | rs761954844 | chr19 | 11110697 | A | G | LDLR | KEEP | KEEP | KEEP | DROP | DROP | 6.91E-04 | 0.0005%~0.001% |
| FH | rs749038326 | chr19 | 11102741 | T | G | LDLR | DROP | KEEP | DROP | DROP | DROP | 0.00E+00 | 0%~0.0001% |
| FH | rs879254996 | chr19 | 11116858 | T | G | LDLR | DROP | KEEP | DROP | DROP | DROP | 0.00E+00 | 0%~0.0001% |
| FH | rs730882109 | chr19 | 11116900 | G | C | LDLR | DROP | KEEP | DROP | DROP | DROP | 0.00E+00 | 0%~0.0001% |
| MODY | rs193922273 | chr7 | 44145266 | T | A | GCK | KEEP | DROP | DROP | DROP | DROP | 0.00E+00 | 0%~0.0001% |
| MODY | rs193922268 | chr7 | 44145593 | G | A | GCK | KEEP | DROP | DROP | DROP | DROP | 0.00E+00 | 0%~0.0001% |
| MODY | rs104894016 | chr7 | 44145618 | T | C | GCK | KEEP | DROP | DROP | DROP | DROP | 0.00E+00 | 0%~0.0001% |
| MODY | rs193922262 | chr7 | 44145636 | A | C | GCK | KEEP | KEEP | KEEP | KEEP | DROP | 0.00E+00 | 0%~0.0001% |
| MODY | rs587780343 | chr7 | 44145638 | A | C | GCK | DROP | KEEP | DROP | DROP | DROP | 0.00E+00 | 0%~0.0001% |
| MODY | rs193922252 | chr7 | 44146479 | TT | CG | GCK | DROP | KEEP | DROP | DROP | DROP | 0.00E+00 | 0%~0.0001% |
| MODY | rs193922336 | chr7 | 44146575 | A | G | GCK | DROP | KEEP | DROP | DROP | DROP | 0.00E+00 | 0%~0.0001% |
| MODY | rs104894011 | chr7 | 44147720 | A | C | GCK | KEEP | KEEP | KEEP | KEEP | DROP | 0.00E+00 | 0%~0.0001% |
| MODY | rs193922312 | chr7 | 44149824 | C | G | GCK | DROP | DROP | DROP | DROP | DROP | 0.00E+00 | 0%~0.0001% |
| MODY | rs886039380 | chr7 | 44150015 | T | C | GCK | DROP | KEEP | DROP | DROP | DROP | 0.00E+00 | 0%~0.0001% |
| MODY | rs193922305 | chr7 | 44150016 | T | C | GCK | DROP | KEEP | DROP | DROP | DROP | 0.00E+00 | 0%~0.0001% |
| MODY | rs193922304 | chr7 | 44150021 | C | G | GCK | KEEP | DROP | DROP | DROP | DROP | 0.00E+00 | 0%~0.0001% |
| MODY | rs193922296 | chr7 | 44150999 | T | C | GCK | DROP | KEEP | DROP | DROP | DROP | 0.00E+00 | 0%~0.0001% |
| MODY | rs193922290 | chr7 | 44152381 | A | T | GCK | KEEP | DROP | DROP | DROP | DROP | 0.00E+00 | 0%~0.0001% |
| MODY | rs794727236 | chr7 | 44153391 | T | C | GCK | DROP | DROP | DROP | DROP | DROP | 0.00E+00 | 0%~0.0001% |
| MODY | rs878853246 | chr7 | 44153397 | A | G | GCK | DROP | KEEP | DROP | DROP | DROP | 0.00E+00 | 0%~0.0001% |
| MODY | rs193922329 | chr7 | 44153433 | A | G | GCK | DROP | KEEP | DROP | DROP | DROP | 0.00E+00 | 0%~0.0001% |
| MODY | rs781260712 | chr7 | 44188908 | A | C |  | KEEP | DROP | DROP | DROP | DROP | 0.00E+00 | 0%~0.0001% |
| MODY | rs193929355 | chr11 | 17387128 | T | C | KCNJ11 | KEEP | KEEP | KEEP | KEEP | DROP | 0.00E+00 | 0%~0.0001% |
| MODY | rs587783673 | chr11 | 17387407 | T | C | KCNJ11 | DROP | KEEP | DROP | DROP | DROP | 0.00E+00 | 0%~0.0001% |
| MODY | rs387906398 | chr11 | 17388225 | A | C | KCNJ11 | DROP | KEEP | DROP | DROP | DROP | 0.00E+00 | 0%~0.0001% |
| MODY | rs137852671 | chr11 | 17394295 | T | C | ABCC8 | DROP | KEEP | DROP | DROP | DROP | 0.00E+00 | 0%~0.0001% |
| MODY | rs28936371 | chr11 | 17394334 | A | G | ABCC8 | DROP | DROP | DROP | DROP | DROP | 0.00E+00 | 0%~0.0001% |
| MODY | rs1057516655 | chr11 | 17394336 | G | GC | ABCC8 | KEEP | KEEP | DROP | DROP | DROP | 0.00E+00 | 0%~0.0001% |
| MODY | rs193922405 | chr11 | 17394360 | T | C | ABCC8 | DROP | KEEP | DROP | DROP | DROP | 0.00E+00 | 0%~0.0001% |
| MODY | rs1057516404 | chr11 | 17395230 | T | C | ABCC8 | DROP | DROP | DROP | DROP | DROP | 0.00E+00 | 0%~0.0001% |
| MODY | rs758844607 | chr11 | 17395260 | A | AG |  | KEEP | DROP | DROP | DROP | DROP | 0.00E+00 | 0%~0.0001% |
| MODY | rs193922402 | chr11 | 17395611 | A | G | ABCC8 | DROP | KEEP | DROP | DROP | DROP | 0.00E+00 | 0%~0.0001% |
| MODY | rs28938469 | chr11 | 17395659 | A | G | ABCC8 | DROP | KEEP | DROP | DROP | DROP | 0.00E+00 | 0%~0.0001% |
| MODY | rs193922401 | chr11 | 17395914 | A | C | ABCC8 | KEEP | KEEP | DROP | DROP | DROP | 0.00E+00 | 0%~0.0001% |
| MODY | rs193922401 | chr11 | 17395914 | A | C |  | KEEP | KEEP | DROP | DROP | DROP | 0.00E+00 | 0%~0.0001% |
| MODY | rs1057517050 | chr11 | 17396914 | A | AC | ABCC8 | DROP | KEEP | DROP | DROP | DROP | 0.00E+00 | 0%~0.0001% |
| MODY | rs766431403 | chr11 | 17397314 | T | C | ABCC8 | DROP | KEEP | DROP | DROP | DROP | 0.00E+00 | 0%~0.0001% |
| MODY | rs367850779 | chr11 | 17402670 | T | C |  | KEEP | KEEP | KEEP | KEEP | DROP | 0.00E+00 | 0%~0.0001% |
| MODY | rs1057516585 | chr11 | 17406942 | T | C | ABCC8 | DROP | DROP | DROP | DROP | DROP | 0.00E+00 | 0%~0.0001% |
| MODY | rs541269678 | chr11 | 17407417 | A | G | ABCC8 | DROP | KEEP | DROP | DROP | DROP | 0.00E+00 | 0%~0.0001% |
| MODY | rs863225278 | chr11 | 17408519 | A | T | ABCC8 | KEEP | KEEP | DROP | DROP | DROP | 0.00E+00 | 0%~0.0001% |
| MODY | rs1057516509 | chr11 | 17432203 | G | C | ABCC8 | DROP | KEEP | DROP | DROP | DROP | 0.00E+00 | 0%~0.0001% |
| MODY | rs372307320 | chr11 | 17442849 | T | C | ABCC8 | DROP | KEEP | DROP | DROP | DROP | 0.00E+00 | 0%~0.0001% |
| MODY | rs193929366 | chr11 | 17461774 | T | G | ABCC8 | KEEP | DROP | DROP | DROP | DROP | 0.00E+00 | 0%~0.0001% |
| MODY | rs770664202 | chr11 | 17463477 | C | CCCAT | | DROP | KEEP | DROP | DROP | DROP | 0.00E+00 | 0%~0.0001% |
| MODY | rs193922593 | chr12 | 120979049 | T | C | HNF1A-AS1;HNF1A | KEEP | KEEP | KEEP | KEEP | DROP | 0.00E+00 | 0%~0.0001% |
| MODY | rs137853243 | chr12 | 120988841 | T | C | HNF1A | DROP | KEEP | DROP | DROP | DROP | 0.00E+00 | 0%~0.0001% |
| MODY | rs771108132 | chr12 | 120994237 | T | C |  | KEEP | KEEP | KEEP | KEEP | DROP | 0.00E+00 | 0%~0.0001% |
| MODY | rs193922604 | chr12 | 120994240 | T | G | HNF1A | DROP | KEEP | DROP | DROP | DROP | 0.00E+00 | 0%~0.0001% |
| MODY | rs137853238 | chr12 | 120994265 | A | G | HNF1A | KEEP | DROP | DROP | DROP | DROP | 0.00E+00 | 0%~0.0001% |
| MODY | rs193922576 | chr12 | 120996557 | G | GC | HNF1A | DROP | KEEP | DROP | DROP | DROP | 0.00E+00 | 0%~0.0001% |
| MODY | rs776793516 | chr12 | 120997491 | G | GCA |  | KEEP | DROP | DROP | DROP | DROP | 0.00E+00 | 0%~0.0001% |
| MODY | rs80356662 | chr13 | 27924381 | A | G | PDX1 | DROP | DROP | DROP | DROP | DROP | 0.00E+00 | 0%~0.0001% |
| MODY | rs121918672 | chr17 | 37731814 | A | G | HNF1B | DROP | DROP | DROP | DROP | DROP | 0.00E+00 | 0%~0.0001% |
| MODY | rs1057517744 | chr17 | 37739443 | A | G | HNF1B | KEEP | KEEP | DROP | DROP | DROP | 0.00E+00 | 0%~0.0001% |
| MODY | rs1800575 | chr17 | 37739455 | A | G | HNF1B | DROP | KEEP | DROP | DROP | DROP | 0.00E+00 | 0%~0.0001% |
| MODY | rs193922476 | chr20 | 44418502 | TCAA | GCT | HNF4A | DROP | KEEP | DROP | DROP | DROP | 0.00E+00 | 0%~0.0001% |
| MODY | rs193922470 | chr20 | 44428458 | C | G | HNF4A | DROP | KEEP | DROP | DROP | DROP | 0.00E+00 | 0%~0.0001% |
| MODY | rs1057521093 | chr7 | 44147810 | C | T | GCK | DROP | KEEP | DROP | DROP | DROP | 0.00E+00 | 0%~0.0001% |
| MODY | rs1554335421 | chr7 | 44149860 | T | C | GCK | DROP | KEEP | DROP | DROP | DROP | 0.00E+00 | 0%~0.0001% |
| MODY | rs80356624 | chr11 | 17387490 | T | C | KCNJ11 | DROP | DROP | DROP | DROP | DROP | 0.00E+00 | 0%~0.0001% |
| MODY | rs1564869850 | chr11 | 17393124 | T | C |  | DROP | KEEP | DROP | DROP | DROP | 0.00E+00 | 0%~0.0001% |
| MODY | rs746480424 | chr11 | 17394333 | T | C | ABCC8 | KEEP | KEEP | DROP | DROP | DROP | 0.00E+00 | 0%~0.0001% |
| MODY | rs151344623 | chr11 | 17397055 | T | C | ABCC8 | DROP | KEEP | DROP | DROP | DROP | 0.00E+00 | 0%~0.0001% |
| MODY | rs193922400 | chr11 | 17404524 | T | C | ABCC8 | DROP | DROP | DROP | DROP | DROP | 0.00E+00 | 0%~0.0001% |
| MODY | rs749271190 | chr11 | 17412665 | T | C | ABCC8 | KEEP | KEEP | KEEP | KEEP | DROP | 0.00E+00 | 0%~0.0001% |
| MODY | rs1463923467 | chr12 | 120994163 | A | G |  | DROP | DROP | DROP | DROP | DROP | 0.00E+00 | 0%~0.0001% |
| MODY |  | chr17 | 37731666 | C | CTG |  | KEEP | DROP | DROP | DROP | DROP | 0.00E+00 | 0%~0.0001% |
| MODY |  | chr17 | 37739437 | TA | T |  | DROP | KEEP | DROP | DROP | DROP | 0.00E+00 | 0%~0.0001% |
| MODY | rs1568670702 | chr17 | 37739545 | A | G |  | DROP | KEEP | DROP | DROP | DROP | 0.00E+00 | 0%~0.0001% |
| MODY | rs886041820 | chr17 | 37739577 | TG | T |  | KEEP | DROP | DROP | DROP | DROP | 0.00E+00 | 0%~0.0001% |
| MODY | rs121918671 | chr17 | 37744584 | A | C | HNF1B | DROP | KEEP | DROP | DROP | DROP | 0.00E+00 | 0%~0.0001% |
| MODY |  | chr17 | 37744653 | A | C |  | DROP | KEEP | DROP | DROP | DROP | 0.00E+00 | 0%~0.0001% |
| MODY | rs1392795567 | chr20 | 44414663 | A | G | HNF4A | DROP | KEEP | DROP | DROP | DROP | 0.00E+00 | 0%~0.0001% |
| Thrombophilia | rs121909566 | chr1 | 173904013 | T | C | SERPINC1 | KEEP | KEEP | KEEP | KEEP | DROP | 0.00E+00 | 0%~0.0001% |
| Thrombophilia | rs121909557 | chr1 | 173904044 | T | C | SERPINC1 | KEEP | KEEP | KEEP | KEEP | DROP | 0.00E+00 | 0%~0.0001% |
| Thrombophilia | rs121909562 | chr1 | 173911942 | A | G | SERPINC1 | KEEP | KEEP | KEEP | KEEP | DROP | 0.00E+00 | 0%~0.0001% |
| Thrombophilia | rs121918155 | chr2 | 127426227 | C | G | PROC | KEEP | KEEP | DROP | DROP | DROP | 0.00E+00 | 0%~0.0001% |
| Thrombophilia | rs121918156 | chr2 | 127427219 | T | C | PROC | DROP | KEEP | DROP | DROP | DROP | 0.00E+00 | 0%~0.0001% |
| Thrombophilia | rs121918154 | chr2 | 127428374 | T | C | PROC | KEEP | KEEP | KEEP | KEEP | DROP | 0.00E+00 | 0%~0.0001% |
| Thrombophilia | rs121918158 | chr2 | 127428575 | A | G | PROC | DROP | KEEP | DROP | DROP | DROP | 0.00E+00 | 0%~0.0001% |
| Thrombophilia | rs121918475 | chr3 | 93898462 | A | G | PROS1 | DROP | KEEP | DROP | DROP | DROP | 0.00E+00 | 0%~0.0001% |
| Thrombophilia | rs137852354 | chrX | 154837677 | A | G | F8 | KEEP | KEEP | DROP | DROP | DROP | 0.00E+00 | 0%~0.0001% |
| Thrombophilia | rs387906457 | chrX | 154904918 | A | T | F8 | KEEP | KEEP | KEEP | KEEP | DROP | 0.00E+00 | 0%~0.0001% |
| Thrombophilia | rs137852432 | chrX | 154947846 | C | G | F8 | KEEP | KEEP | DROP | DROP | DROP | 0.00E+00 | 0%~0.0001% |
| Thrombophilia | rs137852368 | chrX | 154966634 | A | G | F8 | DROP | DROP | DROP | DROP | DROP | 0.00E+00 | 0%~0.0001% |
| Thrombophilia | rs137852397 | chrX | 154987237 | A | C | F8 | DROP | KEEP | DROP | DROP | DROP | 0.00E+00 | 0%~0.0001% |

Dis, disease; SNP, single-nucleotide polymorphism; CHR, chromosome; POS, position; Alt, alternative; Ref, reference; WGS MAF, whole-genome sequencing minor allele frequency.

**Supplemental methods**

**The command of the Rare-het adjustment for Linux**

We used the axiomBestPractices (version 1.2.4) program, which is one of the analysis pipelines executes with Affymetrix^®^ Power Tools (APT), for rare-het adjustment. It is available at the support section of the Affymetrix website ([www.affymetrix.com](http://www.affymetrix.com)). Based on the Axiom Genotyping Solution Data Analysis User Guide (<https://assets.thermofisher.com/TFS-Assets/LSG/manuals/axiom_genotyping_solution_analysis_guide.pdf>), we used following command:

python2 axiomBestPractices-1.2.4/axiomBestPractices.py \

Axiom_TWB_2.na36.r2.a2.p1 \

cel_files.txt \

-o output_dir \

--force \

--do-rare-het-adjustment true

**The command of Advanced normalization for Linux**

The advnorm (version 1.2 ) program is provide by Thermo Fisher Scientific by request. It can automatically select probesets that need to be normalized from the output files of the axiomBestPractices program and complete the adjustment by the following command:

bash advnorm/advnorm.sh \

--summary-file AxiomGT1.summary.txt \

--calls-file AxiomGT1.calls.txt \

--report-file AxiomGT1.report.txt \

--trustcheck-file AxiomGT1.trustcheck.txt \

--analysis-files-path Axiom_TPM.na36.r2.a2.p1 \

--snp-priors-file Axiom_TPM.na36.r2.a2.p1/Axiom_TPM.r2.snp_specific_prior.txt \

--snp-specific-param-file Axiom_TPM.na36.r2.a2.p1/Axiom_TPM.r2.snp_specific_parameters.txt \

--special-snps-file Axiom_TPM.na36.r2.a2.p1/Axiom_TPM.r2.specialSNPs \

--ps2snp-file Axiom_TPM.na36.r2.a2.p1/Axiom_TPM.r2.ps2snp_map.ps \

--output-dir output_dir \

--probeset-ids target_probeset.ps \

To evaluate the commutating times, we use a Dell Poweredge FC830 server equipped with Intel(R) Xeon(R) CPU E5-4620 V3 @ 2.00GHz quad-core processorX4 and 768GB RAM. We used raw data of 30 plates (2,876 samples) as a batch to process. For rare-het adjustment and advanced normalization, it took 11 hr 16 min and 25 hr 38 min to process. Advanced normalization only can use one core of CPU. It also took 1hr30min to compare the results and keep the consistent value. The whole process needs 38hr 24min for 30 plates of samples.

|  | Rare-het adjustment | | Advanced normalization | | Keep consistent values | |
| --- | --- | --- | --- | --- | --- | --- |
| 30plate \| 2876 samples | min | sec | min | sec | min | sec |
| QC | 577 | 31.8 | 1497 | 50.4 |  |  |
| make pedigree file | 0 | 0.7 | 0 | 0.7 |  |  |
| export genotype to vcf | 58 | 12.4 | 0 | 0.7 |  |  |
| select chr | 25 | 6.8 | 24 | 34.6 |  |  |
| rename chr | 7 | 17.8 | 7 | 16.9 |  |  |
| norm vcf | 7 | 59.2 | 7 | 58.6 |  |  |
|  | 674 | 128.6 | 1535 | 162.0 |  |  |
|  |  | **11hr 16min** |  | **25hr 38min** |  |  |
| compare apt and adnorm genotype |  |  |  |  | 89 | 22.2 |
| convert final vcf to plink |  |  |  |  | 0 | 26.9 |
|  |  |  |  |  | 89 | 49.1 |
|  |  |  |  |  |  | **1hr 30min** |
| Total |  |  |  |  |  | **38hr24min** |
